# *Mycobacterium haemophilum* Masquerading as Leprosy in a Renal Transplant Patient

**DOI:** 10.1155/2013/793127

**Published:** 2013-11-28

**Authors:** Nathanial K. Copeland, Navin S. Arora, Tomas M. Ferguson

**Affiliations:** ^1^Department of Medicine, Tripler Army Medical Center, Honolulu, HI 96859, USA; ^2^Dermatology Service, Tripler Army Medical Center, Honolulu, HI 96859, USA; ^3^Infectious Disease Service, Tripler Army Medical Center, Honolulu, HI 96859, USA

## Abstract

Opportunistic infections following immunosuppression in solid organ transplant (SOT) patients are common complications with the skin being a common sight of infection. Nontuberculous mycobacteria (NTM) are rare but potential causes of skin infection in SOT patients. We present a case of an adult male immunosuppressed following renal transplantation who presented with an asymptomatic rash for several months. The patient's skin eruption consisted of erythematous papules and plaques coalescing into an annular formation. After failure of the initial empiric therapy, a punch biopsy was performed that demonstrated nerve involvement suspicious for *Mycobacterium leprae*. However, culture of the biopsy specimen grew acid-fast bacilli that were subsequently identified as *M. haemophilum*. His rash improved after a prolonged course of clarithromycin and ciprofloxacin. Both organisms are potential causes of opportunistic skin infections and can be difficult to distinguish with similar predilection for skin and other biochemical and genetic similarities. Ultimately they can be distinguished with culture as *M. haemophilum* will grow in culture and *M. leprae* will not. This case was unique due to nerve involvement on biopsy which is classically seen on biopsies of leprosy.

## 1. Introduction

Effective immunosuppression has allowed for the development of many life-saving treatments such as organ transplantation, but it has also opened a Pandora's Box of potential side effects including opportunistic infections. A wide variety of unique and often diagnostically challenging infections are the frequent result of the immunosuppression needed following solid organ transplant (SOT). Though opportunistic infections with nontuberculous mycobacteria (NTM) are uncommon in SOT patients, when they do occur, the skin and soft tissue are frequent sites of infection. The incidence for NTM infection in renal transplant patients is estimated at 0.16–0.38% with skin and soft tissue being the most common sites of infection [1]. The following case will present a rash initially thought to be leprosy but subsequently diagnosed as dermatitis due to *Mycobacterium haemophilum*.

## 2. Case Presentation

**Figure 1:**
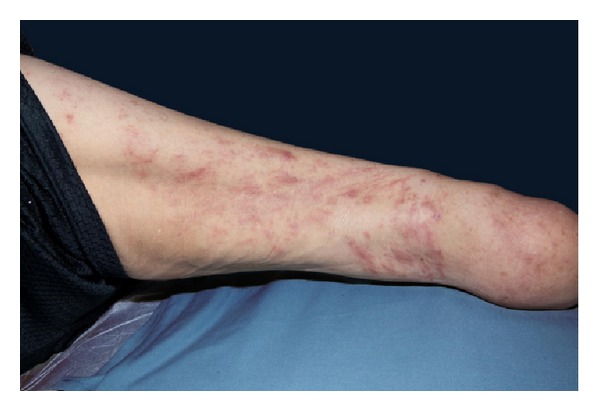
Asymptomatic rash as it appeared following treatment with steroid and antifungal creams characterized by multiple erythematous papules and plaques, some coalescing into an annular pattern.

A 67-year-old male with a history significant for immunosuppression secondary to renal transplantation presented with a 4-month history of an asymptomatic rash and swelling over his right thigh. The eruption developed gradually over several weeks; treatment with an antifungal cream was unsuccessful and a topical corticosteroid cream worsened the eruption. The patient wore a prosthetic limb on his right leg secondary to a below the knee amputation. Of note, the eruption began distally on his residual limb and spread proximally. He had type 2 diabetes mellitus with nephropathy and a cadaveric renal transplant six years earlier. He had been on immunosuppressive therapy since his renal transplant, consisting of a stable dose of cyclosporine 75 mg twice daily, mycophenolic acid 540 mg twice daily, and prednisone 5 mg daily. He had a travel history remarkable for visits to Thailand, Vietnam, and Panama. Cutaneous examination revealed multiple erythematous papules and plaques, some coalescing into an annular pattern on his right thigh (Figure 1).

**Figure 2:**
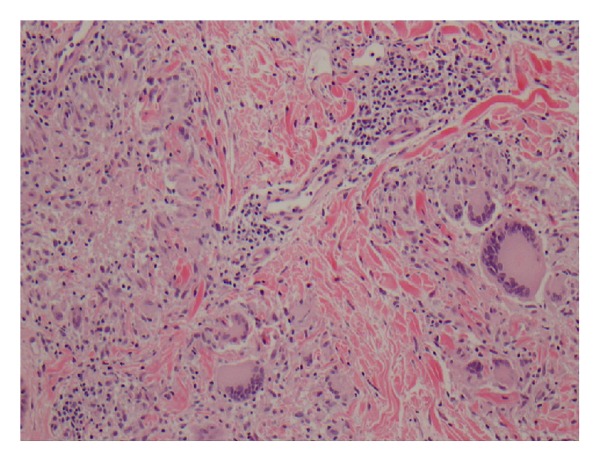
H&E stain demonstrates dermal nonnecrotizing tuberculoid granulomas interspersed with lymphocytes and rare neutrophils.

**Figure 3:**
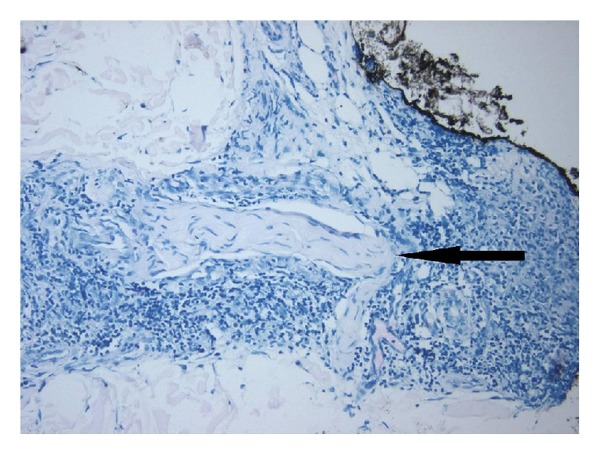
Fite stain revealed areas of peripheral nerve involvement in the granulomatous inflammatory process (arrow).

The patient underwent a punch biopsy for diagnosis. The H&E stain on the biopsy revealed dermal non-necrotizing tuberculoid granulomas interspersed with lymphocytes and rare neutrophils (Figure 2). Acid-fast stain was positive for rare acid-fast bacilli (AFB, not pictured) and revealed granulomatous involvement of a peripheral nerve bundle (Figure 3, arrow) suspicious for *M. leprae*. Period acid-Schiff stain was negative for fungal elements. AFB culture grew mycobacteria which 16s rDNA sequencing confirmed as *M. haemophilum*. The patient was treated with a 6-month course of clarithromycin 500 mg twice daily and ciprofloxacin 500 mg twice daily with complete resolution of the infection and swelling.

## 3. Discussion

*M. haemophilum* is a slow growing NTM that has potential for causing infection, most notably in immunocompromised hosts [1, 2]. *M. haemophilum* has a predilection for the skin due to its optimal growth temperature of 30°C; however, disseminated infections have been reported [1–3]. Like all NTM, *M. haemophilum* is an environmental pathogen with some suggestion of water as a reservoir for the organisms [2]. *M. haemophilum* cutaneous infections manifest as painless erythematous papules, plaques, and nodules that may coalesce into annular formations and can progress to necrotic abscesses and chronic ulcers. Early lesions are typically painless, though they become painful if they progress to abscesses or ulcers [2, 3]. There is a broad differential for the variable skin manifestations including various NTM species, deep fungal infections, cutaneous lymphoma, and the varied spectrum of cutaneous leprosy manifestations. Histopathology often shows granulomatous or mixed suppurative and granulomatous inflammation and AFB are usually present on acid-fast stains, but this pattern is not specific for any species of NTM [4]. Diagnosis of NTM is challenging and requires culture often aided by molecular testing such as rDNA sequencing. The growth of *M. haemophilum* requires incubation at 30°C and addition of iron or hemin to the culture medium [1, 2]. Treatment for *M. haemophilum* typically consists of combination of clarithromycin, ciprofloxacin, and a rifamycin for typically 6 weeks to 6 months and up to 2 years in immunosuppressed patients [2, 3]. The specific regimen may have to be tailored and closely monitored due to the significant potential for drug interactions with immunosuppressive regimens.

This particular case was unique based on nerve bundle involvement, which was highly suggestive of leprosy. *M. leprae* is a very rare cause of skin and soft tissue infections in SOT described in a total of 16 cases worldwide, most of which were in renal transplant patients [5, 6]. This patient was felt to be at increased risk for leprosy due to immunosuppression and travel to several areas with higher prevalence of leprosy [7]. His lesions were clinically consistent with leprosy, and though sensation was intact, up to 30% of leprosy cases will have nonanesthetic lesions [8]. *M. haemophilum* and *M. leprae* have several similarities including predilection for skin infections particularly in distal, cooler locations; comparable cell wall composition; and areas of close genetic homology [2, 8]. The presence of nerve involvement is one of the key histological features to discriminate leprosy from other causes of granulomatous inflammation including other NTM [8]. Ultimately culture was essential in distinguishing these organisms as *M. haemophilum *will grow in culture and *M. leprae* will not [1, 2, 8]. This case is the first known case describing nerve involvement by a NTM other than *M. leprae*.
